# Supplementary figures and images for: High ME1 Expression Is a Molecular Predictor of Post-Transplant Survival of Patients with Acute Myeloid Leukemia
Source: Cancers (Basel). 2022 Dec 31;15(1):296. doi: 10.3390/cancers15010296 (PMC9818450; doi:10.3390/cancers15010296)

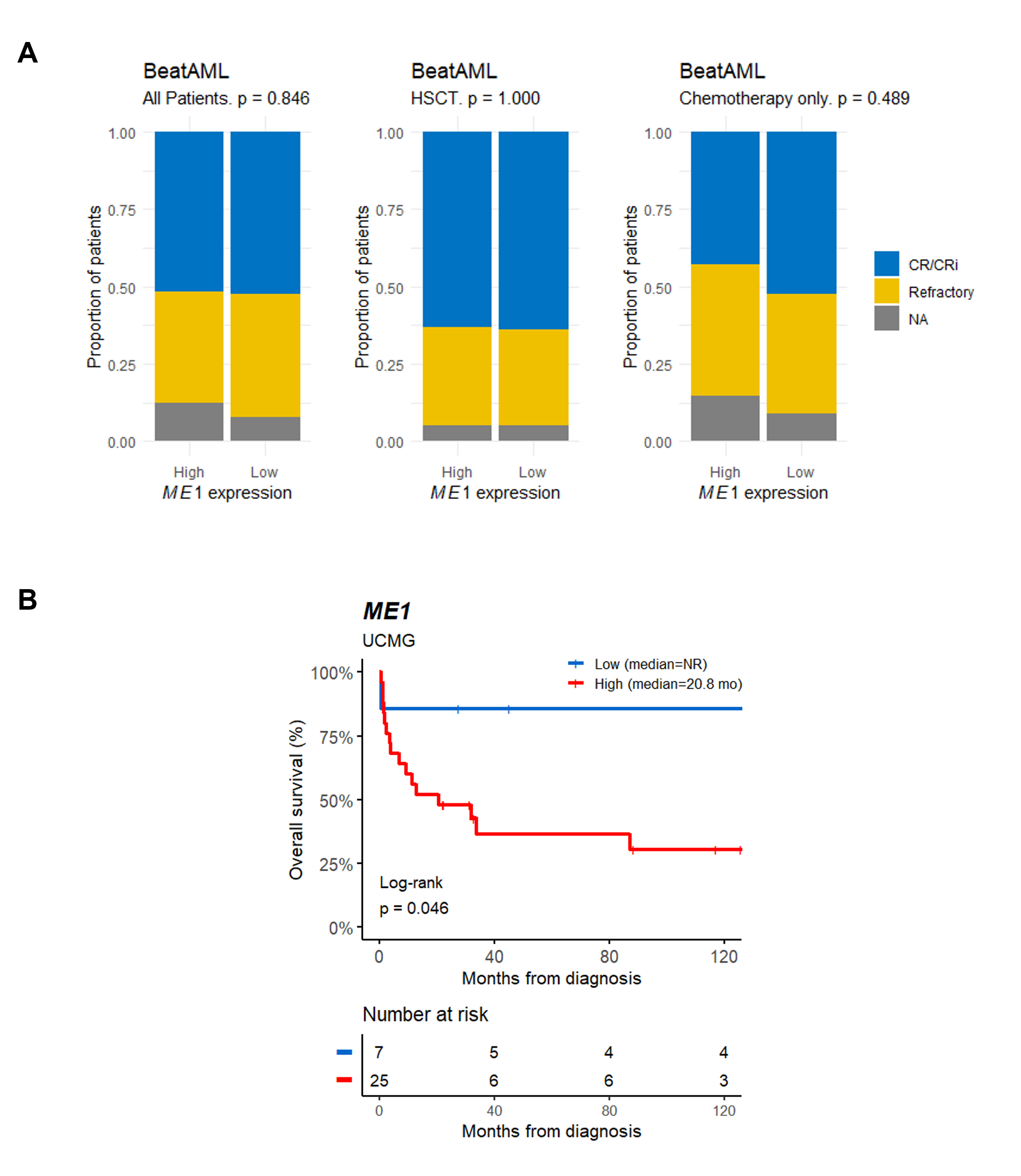

Supplement: Supplementary file 1 [file cancers-15-00296-s001.zip › cancers-1894653-supplementary/Supplementary_Ortiz_et_al/Figure S1.png]

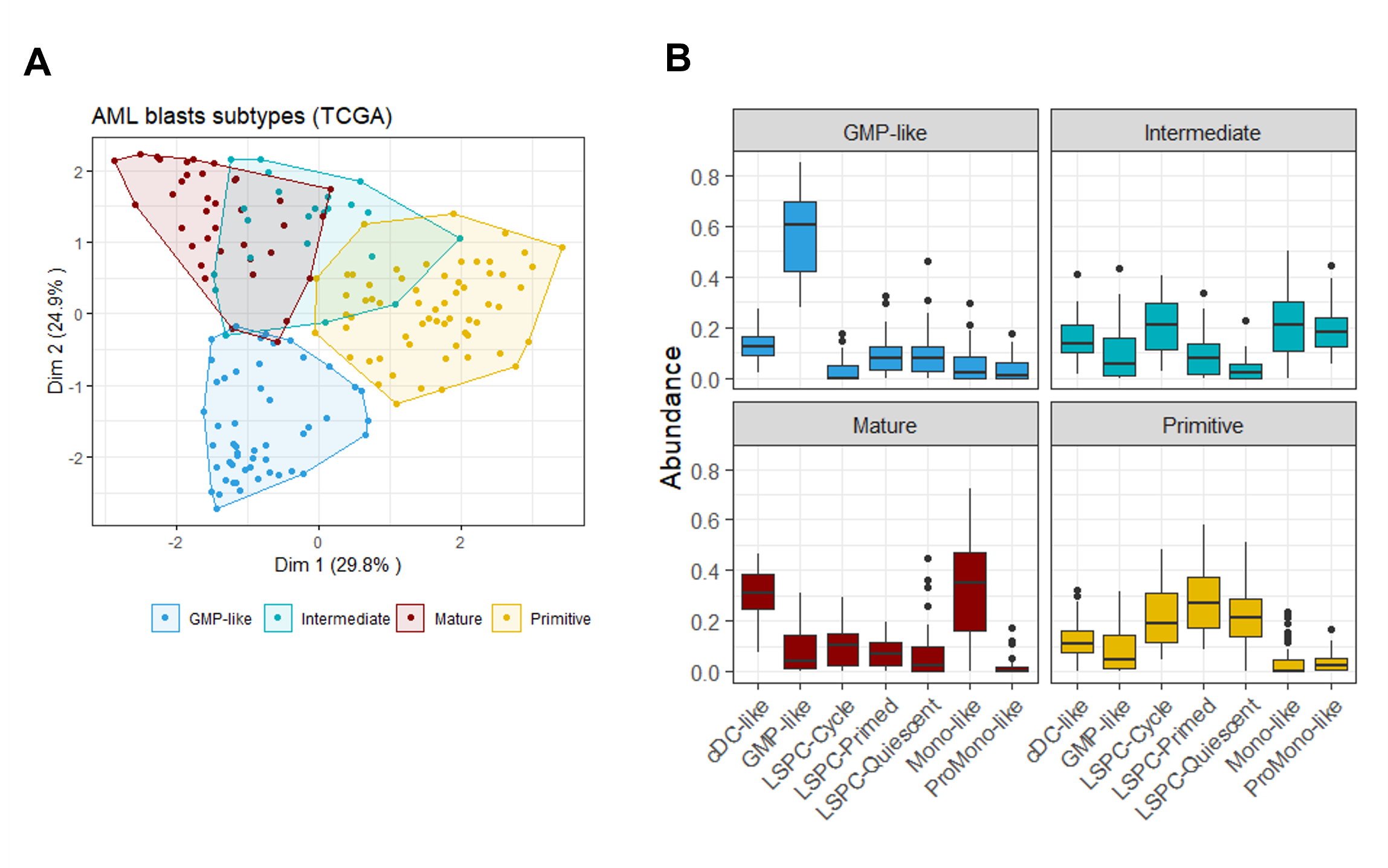

Supplement: Supplementary file 1 [file cancers-15-00296-s001.zip › cancers-1894653-supplementary/Supplementary_Ortiz_et_al/Figure S2.png]

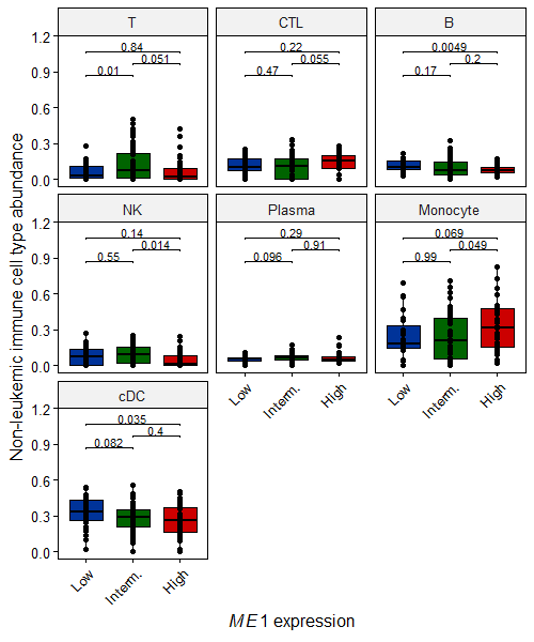

Supplement: Supplementary file 1 [file cancers-15-00296-s001.zip › cancers-1894653-supplementary/Supplementary_Ortiz_et_al/Figure S3.png]

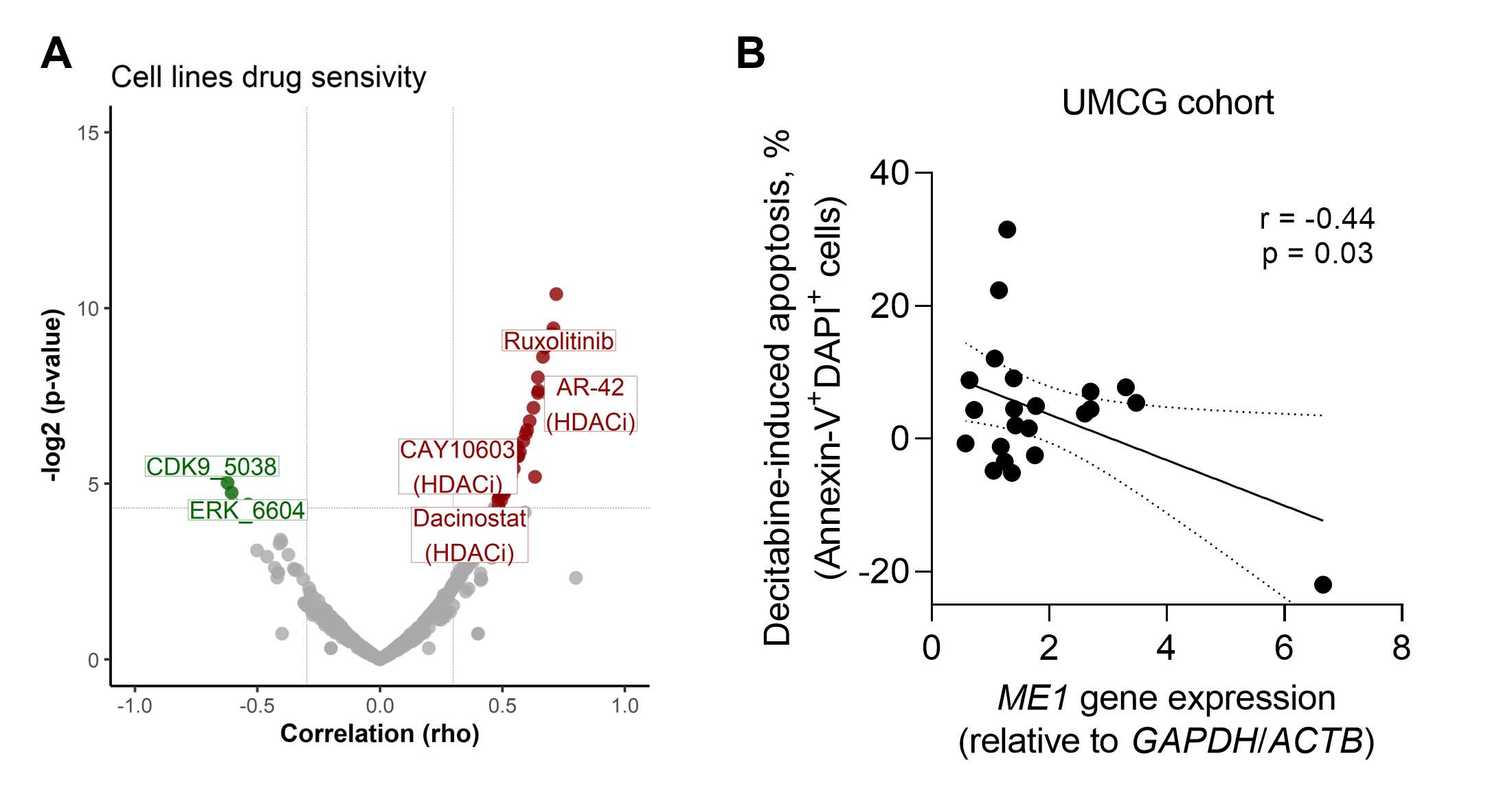

Supplement: Supplementary file 1 [file cancers-15-00296-s001.zip › cancers-1894653-supplementary/Supplementary_Ortiz_et_al/Figure S4.png]

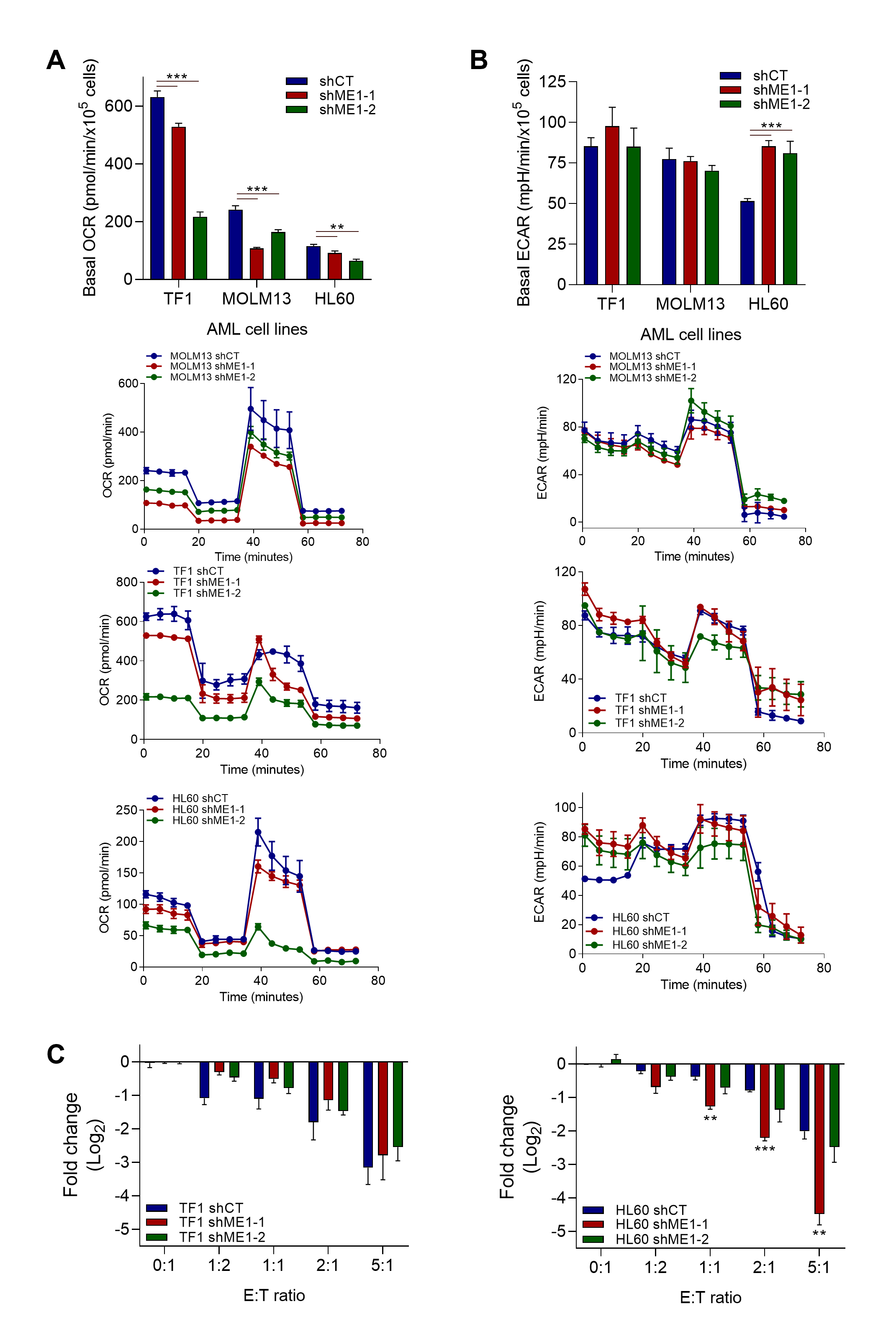

Supplement: Supplementary file 1 [file cancers-15-00296-s001.zip › cancers-1894653-supplementary/Supplementary_Ortiz_et_al/Figure S5.png]

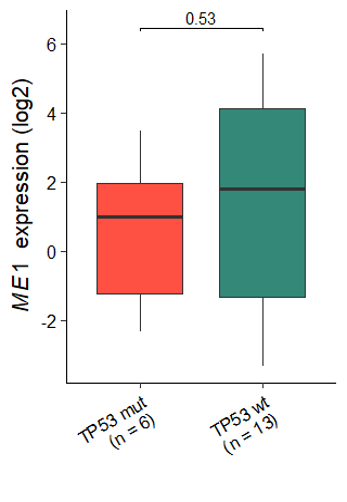

Supplement: Supplementary file 1 [file cancers-15-00296-s001.zip › cancers-1894653-supplementary/Supplementary_Ortiz_et_al/Figure S6.png]
